# Contribution of fortified foods and dietary supplements to total nutrient intakes and their adequacy in Japanese adults

**DOI:** 10.1186/s40795-024-00935-w

**Published:** 2024-09-27

**Authors:** Minami Sugimoto, Keiko Asakura, Nana Shinozaki, Kentaro Murakami, Shizuko Masayasu, Satoshi Sasaki

**Affiliations:** 1 Department of Environmental and Occupational Health, School of Medicine, Toho University, Tokyo, Japan; 2 Institute of Future Initiatives, The University of Tokyo, Tokyo, Japan; 3 Department of Preventive Medicine, School of Medicine, Toho University, Omori-Nishi 5-21-16, Ota-ku, Tokyo, 143-8540 Japan; 4 Department of Nutritional Epidemiology and Behavioural Nutrition, Graduate School of Medicine, The University of Tokyo, Tokyo, Japan; 5 Department of Social and Preventive Epidemiology, School of Public Health, The University of Tokyo, Tokyo, Japan; 6 Ikurien-Naka, Ibaraki, Japan

**Keywords:** Fortified foods, Dietary supplements, Nutrient intake, Dietary reference intakes for Japanese

## Abstract

**Background:**

This study examined how fortified foods and dietary supplements contributed to total nutrient intakes and their adequacy in Japanese adults.

**Methods:**

Nutrient intake was estimated from 4-day dietary records of 392 adults (20–69 years) in total intake considering intakes from fortified foods and dietary supplements and in base diets without considering their intake. Users were defined as participants who used at least one fortified foods and/or dietary supplements during the 4-day recording period. The proportion of participants whose intake was below the Estimated Average Requirements (EAR) or exceeded the Tolerable Upper Intake Level (UL) provided in the Dietary Reference Intakes for Japanese was calculated.

**Results:**

In 122 identified users (31% of participants) of fortified foods and/or dietary supplements, the mean contributions of fortified foods and dietary supplements to total intake were < 4% and < 21%, respectively, for all 25 examined nutrients. Users were more likely to meet the EAR than non-users for six nutrients in the base diets and nine nutrients in the total intake. Among the users, the prevalence of participants below the EAR decreased by ≥ 10% in the total intake compared with the base diet for five nutrients. No nutrient intake from the base diet exceeded the UL in users and non-users; however, vitamin B_6_ intake in 2% of users exceeded the UL of their total intake.

**Conclusion:**

Although the users of fortified foods and/or dietary supplements had better nutrient intake than non-users in base diets, fortified foods and dietary supplements helped the Japanese users achieve adequate intakes of certain nutrients without a risk of excessive intake (except for vitamin B_6_).

**Supplementary Information:**

The online version contains supplementary material available at 10.1186/s40795-024-00935-w.

## Background

Food fortification and supplementation are strategies used to control micronutrient malnutrition [1]. In recent decades, fortified foods and dietary supplements have been used worldwide, including developed countries [2–4]. For example, research from the United States (US) and Finland has shown that the prevalence of dietary supplement use has increased [5–7]. Other studies have shown that more than half of adults use fortified foods at least in some Western countries [4, 8]. Along with the increase in their use, the adverse health effects of dietary supplements [9] and the risk of excessive intake of fortified foods and dietary supplements [10, 11] are growing concerns. Thus, research on the use of fortified foods and dietary supplements is important from a public health perspective.

Many previous studies have reported differences in sociodemographic characteristics between dietary supplement users and non-users. It is widely observed that dietary supplement users tend to be women [5, 10, 12, 13], older [5, 10], and have high educational levels [5, 10]. However, differences between fortified food users and non-users have not been sufficiently examined [3]. Although several studies examined differences in dietary intake between users and non-users of dietary supplements [14–16], the number of studies are still limited, particularly from Asian countries. If users have sufficient nutrient intake profiles, there may be a risk of excess intake and toxicity owing to the use of fortified foods and dietary supplements. However, only a limited number of studies have examined the contribution of nutrient intake from fortified foods and dietary supplements in meeting or exceeding reference values [4, 8, 11, 14, 17]. In addition, these studies were mainly conducted in Western countries. Studies in Asian countries, including Japan, are scarce [3, 12, 13].

Previous studies have suggested that the most commonly used dietary supplements in Japan differ from those used in Western countries. Multivitamins and mineral products are the most common in the US and Australia [5, 10], whereas liquid-type dietary supplements (mainly made with complex ingredients and used for recovery from tiredness) are the most common in Japan [12]. Additionally, mandatory food fortification has not been implemented in Japan. Thus, it is highly likely that the contribution of fortified foods and dietary supplements to the total nutrient intake in the Japanese population differs considerably from that in the Western population. This study aimed to examine the amount of nutrient intake from fortified foods and dietary supplements in users and how fortified food and dietary supplements contribute to the adequacy of nutrient intake in Japanese adults.

## Methods

The current report followed the STROBE-Nut guidelines [18], whose checklist is presented in online Supporting Material 1.

### Study design and participants

This cross-sectional study was a secondary analysis of the dietary intake data collected from healthy Japanese adults aged 20–69 years. Data were collected from 20 study areas (23 of 47 prefectures in Japan) between February 2013 and March 2013 [19, 20]. A detailed description on this study is available elsewhere [19, 20]. Briefly, the primary objective of this survey was to identify food sources of sodium and potassium. First, 199 dietitians working in welfare facilities were recruited as research dietitians supporting the survey. Next, the research dietitians recruited participants from among their co-workers or family members of co-workers, stratified by sex and by five 10-year age groups (20–29, 30–39, 40–49, 50–59, and 60–69 years). The number of participants was targeted to be 20 adults from each study area. The exclusion criteria for recruitment were as follows: (i) licenced dietary or medical provider, (ii) residence in the prefecture or adjacent prefecture in which the facility was located for < 6 months, (iii) individuals who were under diet therapy prescribed by a doctor or dietitian at the time of the study or within 1 year before the study, (iv) pregnant or lactating women, and (v) individuals who had a history of hospitalisation for nutrition education about diabetes. Among 400 recruited participants, 392 adults (196 men and 196 women) completed dietary records and were included in the present analysis. Although the present dataset has small sample size, it has detailed information for both dietary intake and use of fortified foods and dietary supplements in assessment days. In addition, number of assessment days (i.e., four-day) was longer than national dietary survey in Japan (i.e., only one day) [13]. We analysed the present dietary dataset for the abovementioned aim due to unavailability of an ideal data set with detailed information on dietary intake and the use of dietary supplements and fortified foods in longer evaluation days and larger sample sizes among Japanese.

### Ethical approval

This survey was conducted according to the guidelines laid down in the Declaration of Helsinki, and all procedures involving research study participants were approved by the Ethics Committee of the University of Tokyo, Faculty of Medicine (approval number: 10005; approval date: 7 January, 2013). Written informed consent was obtained from all the participants. This secondary analysis was conducted with the approval of the Ethics Committee of Toho University, School of Medicine (approval number: A23086; approval date: 20 December, 2023). According to the Japanese ethics guidelines, we provided ethical information and a proposal for this study on the website of the Department of Social and Preventive Epidemiology, the University of Tokyo (https://www.nebn.m.u-tokyo.ac.jp/#news) and opportunities to opt-out to the participants. As a result, no participants opted out.

### Dietary record

Dietary intake was assessed using 4 non-consecutive-day dietary records, with 3 working days and 1 day off. Each participant was asked to weigh and record all food, beverages, and dietary supplements consumed on the 4 assessment days, using the provided equipment (e.g. a digital kitchen scale, measuring spoon, and measuring cup) and a recording sheet. When weighing was difficult (e.g. eating out), the restaurant’s name, name of the dishes, and estimated amount of leftovers were reported. All recorded foods and beverages were assigned food item numbers according to the Standard Tables of Food Composition in Japan, Fifth Revised and Enlarged Edition [21], as this was the latest version at the time of data collection. All records were checked by research dietitians at each facility and trained dietitian staff at the survey centre. If needed, the research dietitians contacted the participants to clarify any ambiguities or missing data on the recording sheets.

Information on the use and intake amount of fortified foods and dietary supplements was derived from 4 non-consecutive-day dietary records.

### Dietary intake from base diets

Daily intakes of energy and nutrients from non-fortified foods and non-dietary supplements (hereafter referred to as the “base diet”) were estimated based on dietary records and the Standard Tables of Food Composition in Japan, Seventh Revised and Enlarged Edition [22]. In the Seventh Revised and Enlarged Edition, the same food codes were used as those in the Fifth Revised and Enlarged Edition for almost all foods; however, the composition data were updated. When food codes were changed from the Fifth Revised and Enlarged Edition, the food codes in the dietary data were substituted with another code for the corresponding foods in the Seventh Revised and Enlarged Edition.

Food intake was calculated based on the dietary records. Definitions of the food groups are shown in Supplemental Table 1. The intake of dietary supplements was excluded from the intake of any food group, but the intake of fortified foods was included. For example, fortified milk intake was included in the intake of “milk and dairy food products”.

The daily intakes of energy, nutrients, and food from the base diet were calculated for each assessment day. Furthermore, the average nutrient and food intakes over the 4 assessment days were calculated.

### Dietary intake from fortified foods and dietary supplements

There is no clear definition of fortified foods and dietary supplements in Japan. For this analysis, fortified foods were defined as commonly consumed, processed products in which one or more vitamins and/or minerals were added [1]. We could not find a more detailed definition of fortified foods. Thus, for clarification, we added a further definition: fortified foods are products whose corresponding non-fortified foods are marketed, such as non-fortified rice, flour, milk, and confectionery. Dietary supplements are defined as products whose purpose is to supplement the normal diet, and they are concentrated sources of nutrients or other substances with nutritional or physiological effects, alone or in combination, marketed in dosage form, namely, capsules, pastilles, tablets, pills and other similar forms, sachets of powder, ampoules of liquids, drop dispensing bottles, and other similar forms of liquids and powders designed to be taken in measured small unit quantities [23]. Dietary supplements can include one or more of the following dietary ingredients: (a) vitamins; (b) minerals; (c) herbs and other botanicals; (d) an amino acid; (e) a dietary substance for use by humans to supplement the diet by increasing the total dietary intake; and (f) a concentrate, metabolite, constituent, extract, or combination of any ingredient described in clause (a), (b), (c), (d), or (e) [24]. The definition of dietary supplement by the Dietary Supplement Health and Education Act of 1994 [24] does not include medicine. However, we included non-prescribed medicine as a dietary supplement if the products reported in the dietary record as “dietary supplements” by the participants because some products (such as liquid-type products) were approved as medicine by the Minister of Health, Labour and Welfare, were widely used among Japanese as “dietary supplements” [12]. The brand names of the fortified foods and dietary supplements were identified from the dietary records. Since it was difficult to classify liquid-form products into fortified foods, dietary supplements or general foods (i.e., non-fortified food and non-dietary supplement) according to the abovementioned definition alone, we classified them as described below. First, a sweetened beverage was classified as a dietary supplement when the product was added vitamins or minerals and was approved as medicine by the Minister of Health, Labour and Welfare. A sweetened beverage was classified as a fortified food when the product was added vitamins or minerals but not approved medicine. Otherwise, sweetened beverages were classified as general foods. Botanical juices (e.g. *Peucedanum japonicum* juice) or fermented beverages (e.g. black vinegar beverage) were classified as dietary supplements when the product featured the main ingredients (i.e. botanical or fermented substances) but not vitamins or minerals and when it was not typical to consume the main ingredients in the form of drinks. The first author (M. S.) checked the dietary records and identified and classified the products as fortified foods, dietary supplements, or general foods. When doubts arose, decisions were made in consultation with the second author (K.A.).

The authors developed new food composition tables for fortified foods and dietary supplements based on nutrient content information on manufacturers’ websites because the Standard Tables of Food Composition in Japan do not include their compositions. For fortified foods and dietary supplements not approved as medicine by the Minister of Health, Labour and Welfare, the following information was obtained because they were mandatorily indicated on the food package in Japan: energy (kcal), protein (g), total fat (g), carbohydrate (g), and sodium (salt-g) [25]. Information on other nutrients, such as vitamins and minerals, was obtained, if available. For fortified foods, the composition of nutrients that was not written on the manufacturer’s website was substituted with that of similar non-fortified foods after standardising the product’s energy content level. For example, the potassium content of fortified milk was substituted with that of low-fat milk (food code 13005). For dietary supplements, the content of other nutrients was assumed to be zero when not indicated on the package or the manufacturer’s website. For dietary supplements approved as medicines by the Minister of Health, Labour and Welfare, energy, macronutrients, and sodium content could not be obtained from the product label or the manufacturer’s website. Thus, the macronutrient content was substituted with the content of foods used as pharmaceutical additives. For example, when sugar was used as a pharmaceutical additive for a 2-g tablet-type product, its macronutrient content was substituted with sugar with the same weight as the products (i.e. 2 g of sugar). When sugar was used as a pharmaceutical additive for a drink-type product, its macronutrient content was substituted with the sugar-sweetened beverage content (food code 16052). Otherwise, the content of similar products not approved as medicines were used. For the dietary supplement “*Ao-jiru* (i.e. kale powder)”, food code 16,056 “*Ao-jiru*,* kale*,* powder product*” was assigned because this code and composition data were added from the Standard Tables of Food Composition in Japan, Seventh Revised and Enlarged Edition [22].

Energy and nutrient intakes from fortified foods and dietary supplements were calculated using the intake or dose recorded in the dietary record and the abovementioned food composition table. The daily intake of energy and nutrients on each assessment day was calculated for fortified foods, dietary supplements, and all consumed foods and drinks (i.e. non-fortified foods, non-dietary supplements, fortified foods, and dietary supplements; hereafter referred to as the “total intake”). The average intake over the 4 assessment days was calculated. In addition, the contribution was calculated as percentages of energy and nutrient intake from fortified foods and dietary supplements, respectively, in relation to the intake from all consumed foods and drinks (i.e. the total intake) and the average intake for the 4 assessment days. For example, the percentage contribution of vitamin C from fortified foods was calculated as the average intake of vitamin C from fortified foods for 4 days divided by vitamin C from all consumed foods and drinks for 4 days.

### User of fortified foods and/or dietary supplements

Users of fortified foods and/or dietary supplements were defined as those who used at least one fortified food and/or dietary supplement at least one day during the 4 recording days. Products were categorised according to their product names and ingredients (Table 1) because there is no standard classification for fortified foods and dietary supplements. These categories were defined based on dietary substances featuring brand names or information on the manufacturer’s website. First, products were classified into “nutrient-featured supplements” or “non-nutrient substance-featured supplements”. Nutrient-featured supplements mainly focus on vitamins, minerals, and other nutrients (e.g. amino acids, fats, and dietary fibres). Nutrient-featured supplements were further classified as “multivitamin/mineral supplements”, “single vitamin/mineral supplements”, and “other nutrient supplements”. The multivitamin/mineral products group included “multivitamin and mineral” defined as products including ≥ 3 vitamins and ≥ 1 minerals [26], “multivitamin” containing ≥ 3 vitamins, and “multimineral” containing ≥ 3 minerals. Other products featuring < 3 vitamins or minerals were categorised into the single-vitamin/mineral supplement product group. When the featured substances were nutrients other than vitamins and minerals, they were categorised as other nutrient products. Non-nutrient substance featured supplements were further classified into four groups: (1) “other extract-based supplements”, which were based on microorganisms or a metabolite; constituent; or extract of plant, animal, or microorganisms (e.g. yeast tablet, garlic extract capsule, placenta extract tablet, probiotic products); (2) “food processed supplements”, which were concentrated, dried, juiced, or powdered form of plant or other foods (e.g. bee larva capsule, rice bran tablet, and kale powder); (3) “traditional Chinese medicine” (e.g. *Dang Gui Shao Yao San* powder); and (4) “other unknown supplements”. When the details of these products were unknown owing to insufficient information in the dietary record, the products were categorised as “other unknown supplements”. After classification, the number of users and days of use were calculated using product categories.

**Table 1:** Prevalence of fortified foods and dietary supplements and frequency of use in 4-day dietary record among 392 Japanese adults (20–69 y)

	Number of products	Number of users (*n* = 122)*	% of total participants	Number of days with ≥ 1 use (over 4 recording days)							
				4 days		3 days		2 days		1 day	
				Number of users	% among users	Number of users	% among users	Number of users	% among users	Number of users	% among users
Fortified foods†	24	41	(10)	6	(15)	6	(15)	6	(15)	23	(56)
Any supplements	118	94	(24)	41	(44)	13	(14)	17	(18)	23	(24)
Nutrient featured supplements	47	51	(13)	14	(27)	5	(10)	12	(24)	20	(39)
Multivitamin and mineral supplements‡	5	6	(2)	1	(17)	1	(17)	1	(17)	3	(50)
Single vitamin/mineral supplements§	34	41	(10)	12	(29)	3	(7)	8	(20)	18	(44)
Other nutrient supplements ||	8	9	(2)	2	(22)	0	(0)	3	(33)	4	(44)
Other types of supplements	71	62	(16)	34	(55)	10	(16)	5	(8)	13	(21)
Other extract-based supplements¶	43	43	(11)	25	(58)	7	(16)	5	(12)	6	(14)
Food processed supplements **	21	23	(6)	13	(57)	3	(13)	2	(9)	5	(22)
Traditional Chinese medicine	4	5	(1)	2	(40)	0	(0)	1	(20)	2	(40)
Other unknown supplements††	3	2	(1)	2	(100)	0	(0)	0	(0)	0	(0)

*Some users consumed > 1 type of fortified foods and/or dietary supplements

†Products (cereals, dairy products, confectionery, and beverages) in which one or more vitamins and/or minerals were added

‡Products contain ≥ 3 vitamins or ≥ 3minerals

§Products contain less than 3 vitamins and minerals. Including vitamin B complex with/without other dietary substances

||Products based on nutrients other than vitamins and minerals such as protein, amino acid, and fat

¶Products based on microorganisms or a metabolite, constituent, or extract of plant, animal or microorganisms. (e.g., yeast tablet, garlic extract capsule, placenta extract tablet, probiotic products)

**Products that were concentrated, dried, or powdered form of plant or other foods (e.g., bee larva capsule, rice bran tablet, and kale powder)

††The details of these products were unknown due to insufficient information in the dietary record

### Usual intake calculation

Multiple source method (MSM) [27, 28] was used to calculate the usual intake of energy and nutrients from the base diet and total intake. For each participant, 4-day measurements of energy and nutrient intake were imported into the MSM program. For the base diet, 4-day measurements of energy and nutrient intake from non-fortified and non-dietary supplemented foods were imported. For the total intake, 4-day measurements of energy and nutrient intake from all foods, including fortified foods and dietary supplements, were imported. In the MSM, the usual intake of each participant was calculated using the following three steps: (1) calculating the probability of eating a certain nutrient on a random day for each individual, (2) estimating the usual amount of nutrient intake on a consumption day, and (3) multiplying the resulting numbers from the former two steps by each other. The usual nutrient intake was calculated separately for users and non-users to avoid large day-to-day variations in the intake of fortified foods and dietary supplements among users, affecting the estimation of the usual intake among non-users and vice versa.

### Assessment of nutrient intake inadequacy

The estimated usual nutrient intake was compared with age- and sex-specific reference values in the Dietary Reference Intakes for Japanese, version 2020 [29]. The Estimated Average Requirement (EAR) was defined as “the estimated intake amount that meets the requirements of 50% of the individuals belonging to an age or sex group” [29]. EAR was defined for the following 14 nutrients: protein, vitamin A (retinol equivalents), thiamine, riboflavin, niacin (niacin equivalent), vitamin B_6_, vitamin B_12_, folate, vitamin C, sodium, calcium, magnesium, zinc, and copper. The Tolerable Upper Intake Level (UL) was defined as “the highest average daily nutrient intake level that was unlikely to pose any risk of adverse health effects” [29]. UL was defined for the following 11 nutrients: vitamin A (retinol equivalents), vitamin D, vitamin E (mg/d), niacin (niacin equivalent), folate, calcium, phosphorus, iron, zinc, and copper. For the 14 nutrients with the EAR, participants whose intake was lower than the EAR were considered to have inadequate intake. For the 11 nutrients with the UL, participants whose intake exceeded the UL were considered to have inadequate intake. Reference values are presented in Supplemental Table 2. Niacin intake in niacin equivalent was calculated based on niacin intake (mg) and protein intake by the following formula to consider the amount of niacin biosynthesised from tryptophan: niacin (mg) + protein (mg)/6000. For the EAR of iron, the reference values for menstruating women aged 20–64 years were used.

We excluded biotin, iodine, selenium, chromium, and molybdenum, for which EAR was defined from the current analysis because of the insufficiency of food composition tables for these nutrients in Japan.

### Measurement of other variables

Age and sex were assessed using questionnaires. Body height and weight were measured by a research dietitian or medical staff member at the welfare facility where the research dietitian worked. Body mass index was calculated based on the measured body weight (in kg) divided by the square of the measured body height (in m). Blood pressure was measured by research dietitians, medical staff at the welfare facility, or the participants themselves using sphygmomanometers in welfare facilities. The participants’ past medical history, current medication use, occupation, educational background, and smoking habits were assessed using a questionnaire.

### Statistical analysis

All statistical analyses were performed using the SAS software (version 9.4; SAS Institute Inc.). Statistical tests were two-sided, and statistical significance was set at *P* < 0.05.

Values are presented as mean and standard deviation for continuous variables and as number of participants (%) for categorical variables. We conducted unadjusted between-group comparisons because the aim of this study was to describe nutrition intakes and prevalence of inadequate intake in both users and non-users, and to compare them between two groups. From the public health perspective, unadjusted results would be more useful than adjusted results for this study. The basic characteristics of users and non-users of fortified foods and/or dietary supplements were compared using the two-sample *t*-test for continuous variables and the chi-squared test for categorical variables. Next, the mean intakes of energy and 25 nutrients during the average of 4 recording days from base diet and mean total intake of energy and nutrients among users were compared with those from the total intake (= base diet) among non-users using the two-sample *t*-test. Furthermore, the mean usual intakes of energy and 17 nutrients from base diets and mean usual total intakes of them among users were compared with those among non-users using the two-sample *t*-test. The percentage of participants with an intake below the EAR and exceeding the UL was compared between users and non-users using the chi-squared test, respectively. Furthermore, the mean food intakes of users and non-users were compared using the two-sample *t*-test.

## Results

### Number of users and frequency of use of fortified foods and dietary supplements

All 392 participants completed four-day dietary record and questionnaire. No participants excluded due to incorrectly filling the questionnaire. Among the 392 participants, 122 (31%) were identified as users of fortified foods and/or dietary supplements (Table 1). The number of fortified foods users was 41 (10%) and that of dietary supplements users was 94 (24%).

The most commonly used fortified food was fortified milk (15 users), followed by sweetened beverages (9 users) and fortified rice (6 users). The most commonly used dietary supplements were vitamin B complex with herbal extract/amino acids (mostly taurine) (27 users; liquid- or tablet-type products for recovery from tiredness or health promotion) and glucosamine/chondroitin (12 users). Nutrient-featured supplements were used by 51 participants, and non-nutrient-featured supplements were used by 62 participants. Half of the fortified food users (56% of 41 users) used fortified foods for only 1 day during the 4 recording days. In contrast, 44% of 94 dietary supplement users used any supplements daily for 4 recording days.

### User characteristics

Users, who used at least one fortified foods and/or dietary supplements during the 4-day recording period, were older, had lower body height, and had higher diastolic blood pressure than non-users (Table 2). There were no significant differences in body weight, body mass index, systolic blood pressure, sex, smoking habits, past medical history, current medication use, educational background, or occupation between the users and non-users.

**Table 2:** Characteristics of fortified foods and/or dietary supplements users and non-users among 392 Japanese adults (aged 20–69 y)

	Users (*n* = 122)		Non-users (*n* = 270)		P*
	Mean	SD	Mean	SD	
Age (years)	46.4	14.0	43.7	13.0	0.048
Body height (cm)	162.6	8.8	164.5	8.2	0.03
Body weight (kg)	62.7	13.2	63.0	12.4	0.85
Body mass index (kg/m^2^)	23.6	3.9	23.2	3.5	0.24
Systolic blood pressure (mmHg)†	124.0	14.2	123.3	15.2	0.60
Diastolic blood pressure (mmHg)†	79.7	10.4	77.2	11.5	0.04
	N	(%)	N	(%)	
Sex					0.38
Men	57	(47)	139	(51)	
Women	65	(53)	131	(49)	
Smoking habits					0.59
Never	73	(60)	147	(54)	
Ex-smokers	21	(17)	50	(19)	
Current smokers	28	(23)	73	(27)	
Past medical history					
Any disease	40	(33)	69	(26)	0.14
Hypertension	16	(13)	31	(11)	0.64
Hyperlipidaemia	12	(10)	24	(9)	0.76
Current prescribed medication use					
Yes	37	(30)	58	(21)	0.06
Educational background					0.54
Junior high school	5	(4)	5	(2)	
Senior high school	31	(25)	73	(27)	
Vocational school or junior college	47	(39)	97	(36)	
University or graduate school	39	(32)	95	(35)	
Occupation					0.54
Clerical	44	(36)	120	(44)	
Nursing care	57	(47)	107	(40)	
Medical assistant	3	(2)	9	(3)	
Cooking assistant	9	(7)	15	(6)	
Others	9	(7)	19	(7)	

*P value by t-test for continuous variables and chi-squared test for categorical variables. *P* < 0.05 was considered as significance

† One non-user missing values of blood pressure was excluded from the calculation

### Contribution to nutrient intake from fortified foods and dietary supplements

In a comparison of the base diet between users of fortified foods and/or dietary supplements and non-users (Table 3), users had a higher mean intake of dietary fibre, vitamin D, vitamin E, thiamine, riboflavin, vitamin B_6_, folate, pantothenic acid, vitamin C, potassium, calcium, magnesium, phosphorus, iron, and copper than non-users. In addition, the mean intake of vitamin K and niacin among users was higher than that among non-users after considering the intake of fortified foods and dietary supplements (i.e., total intake). The mean percentage contributions of fortified foods were small (i.e. <5%) for all nutrients, ranging from 0.094% (vitamin K) to 3.9% (vitamin C). The mean percentage contributions of dietary supplements to total nutrient intakes among users varied in terms of nutrients, ranging from 0.01% (saturated fat) to 20.6% (vitamin B_6_). Dietary supplements had a small (< 5%) contribution to the intake of most nutrients but a relatively high contribution to the intake of thiamine (17.9% on average of users), riboflavin (18.3%), and vitamin B_6_ (20.6%).

**Table 3:** Comparison of energy and nutrient intake from base diet, supplement, and fortified foods among fortified foods and/or dietary supplements users and non-users assessed by 4-day diet record among 392 Japanese adults (aged 20–69 y)*

	Users (*n* = 122)															Non-users (*n* = 270)	
	Total intake†		*P*‡	Base diet†		*P*§	Fortified foods		Fortified foods % of total intake||		Dietary supplements		Dietary supplements % of total intake||			Total intake (Base diet)†	
	Mean	SD		Mean	SD		Mean	SD	Mean	SD	Mean	SD	Mean	SD		Mean	SD
Energy (kcal/day)	2173	493	0.27	2151	488	0.48	10	22	0.46	1.1	11	20	0.52	0.93		2114	486
Protein (g/day)	78.2	19.6	0.01	77.1	19.3	0.05	0.42	1.45	0.54	1.9	0.7	2.5	0.80	2.8		73.1	17.9
Total fat (g/day)	68.4	20.7	0.39	68.1	20.6	0.47	0.17	0.53	0.24	0.68	0.14	0.29	0.19	0.43		66.5	19.0
Saturated fat (g/day)	19.8	6.5	0.27	19.7	6.5	0.35	0.11	0.37	0.49	1.4	0.001	0.004	0.01	0.02		19.1	6.3
Carbohydrate (g/day)	287	70	0.28	283	69	0.54	1.7	3.8	0.58	1.3	1.8	3.1	0.64	1.0		279	69
Dietary fiber (g/day)	15.3	5.6	< 0.001	15.2	5.6	< 0.001	0.019	0.09	0.11	0.54	0.12	0.32	0.82	2.5		13.5	4.2
Vitamin A (µg RAE/d)	653	824	0.19	630	820	0.31	4.7	18.1	0.86	3.3	18	91	2.2	9.2		550	667
Vitamin D (µg/d)	9.0	6.3	0.002	8.5	5.9	0.02	0.33	1.9	2.4	8.9	0.2	0.7	1.5	5.9		7.1	5.0
Vitamin E (mg/d)	15.5	48.4	0.005	8.0	2.8	0.004	0.038	0.13	0.45	1.4	7.5	48.1	8.9	21.9		7.3	2.1
Vitamin K (mg/d)	263	123	0.02	258	120	0.05	0.23	0.9	0.094	0.38	5.0	15.8	1.7	5.9		234	111
Thiamin (mg/d)	2.7	7.5	< 0.001	1.03	0.30	0.049	0.030	0.1	1.9	7.4	1.6	7.4	17.9	30.2		0.97	0.29
Riboflavin (mg/d)	2.8	4.8	< 0.001	1.4	0.4	0.01	0.038	0.1	2.0	5.8	1.4	4.8	18.3	28.1		1.3	0.4
Niacin (mgNE/d)¶	22.9	11.1	< 0.001	19.3	6.1	0.29	0.28	1.0	1.1	4.2	3.3	7.9	9.6	17.4		18.6	6.0
Vitamin B_6_ (mg/d)	4.6	12.5	< 0.001	1.4	0.4	< 0.001	0.059	0.24	2.3	7.9	3.1	12.5	20.6	31.6		1.2	0.4
Vitamin B_12_ (µg/d)	15.3	89.7	0.10	6.9	4.7	0.17	0.062	0.26	1.0	4.5	8.4	89.6	3.2	13.4		6.2	4.5
Folate (µg/d)	412	171	< 0.001	396	162	0.01	6.2	25.9	1.3	4.8	10	33	1.9	5.6		356	141
Pantothenic acid (mg/d)	7.2	5.1	< 0.001	6.3	1.7	0.006	0.12	0.3	1.7	4.8	0.7	4.4	3.0	12.5		5.8	1.6
Vitamin C (mg/d)	188	216	< 0.001	125	60	0.001	7.8	31.4	3.9	13.0	55	206	10.4	23.0		106	48
Sodium (salt g/day)	10.5	3.2	0.15	10.5	3.2	0.20	0.016	0.051	0.17	0.63	0.03	0.18	0.31	1.8		10.1	2.8
Potassium (mg/day)	2843	874	< 0.001	2815	863	0.003	16	70	0.50	2.2	12	39	0.39	1.3		2570	683
Calcium (mg/d)	589	233	< 0.001	544	200	0.006	29	100	3.4	10.4	16	59	2.2	6.9		490	167
Magnesium (mg/d)	305	102	0.01	301	98	0.03	1.2	4.7	0.40	1.61	2.8	16.1	0.68	3.3		280	83
Phosphorus (mg/d)	1146	289	0.002	1133	285	0.008	12	45	0.94	3.73	1.9	8.9	0.14	0.53		1056	254
Iron (mg/d)	9.1	2.8	< 0.001	8.8	2.6	0.01	0.25	1.0	2.2	7.5	0.09	0.42	0.94	4.1		8.1	2.2
Zinc (mg/d)	9.0	2.4	0.06	8.8	2.3	0.15	0.046	0.19	0.48	2.0	0.07	0.31	0.75	3.6		8.5	2.6
Copper (mg/d)	1.3	0.4	0.004	1.3	0.4	0.01	0.0018	0.006	0.14	0.47	0.003	0.023	0.28	2.0		1.2	0.3

NE, Niacin Equivalent; RAE, Retinol Active equivalent

*Values were based on the average intake for four assessment days

†Total intake is a sum of intake from the base diet (i.e., intake from non-fortified and non-dietary supplements), fortified foods, and dietary supplements. Total intake among non-users was the same as intake from base diets

‡P-value by t-test between total intake among users and total intake (= intake from base diet) among non-users. *P* < 0.05 was considered as significant

§P-value by t-test between intake from base diet among users and non-users. *P* < 0.05 was considered as significant

|| The % of the contribution of fortified foods [or dietary supplements] = (the average intake amount from fortified foods [or dietary supplements] for four assessment days)/ the average intake amount from all consumed foods, including fortified foods and dietary supplements [i.e., total intake])×100

¶Niacine equivalents = niacin (mg) + protein (mg)/6000

### Contribution to nutrient intake adequacy

In the base diet, the prevalence of participants below the EAR was lower in users of fortified foods and/or dietary supplements for six out of the 14 examined nutrients (vitamins A and B_6_, thiamine, riboflavin, calcium, and zinc) than in non-users (Table 4). In the total intake, the prevalence of participants below the EAR in users was lower than that in non-users for three additional nutrients, namely, vitamin C, magnesium, and iron. Among users, the prevalence of inadequate intake was decreased by ≥ 10% for thiamine, riboflavin, vitamin B_6_, vitamin C, and calcium for the total intake compared with the prevalence for the base diet. However, even for the total intake, the prevalence of inadequacy was > 30% for vitamin A, calcium, magnesium, and iron.

**Table 4:** Comparison of usual energy and nutrient intake from base diet, supplement, and fortified foods among fortified foods and/or dietary supplements﻿ users and non-users assessed by 4-day diet record among 392 Japanese adults (aged 20–69 y)*

	Users†																Non-users					
	Total intake‡ (*n* = 122)								Base diet (*n* = 122)								Total intake (Base diet)‡ (*n* = 270)					
	Mean	SD	Min	Max	< EAR (%)	P§	> UL (%)		Mean	SD	Min	Max	< EAR (%)	P||	> UL (%)		Mean	SD	Min	Max	< EAR (%)	> UL (%)
Energy (kcal/day)	2173	438	1173	3733	-	-	-		2152	432	1168	3668	-	-	-		2114	428	978	3329	-	-
Protein (g/d)	78.2	17.0	34.9	132.4	1	0.33	-		77.1	16.7	34.6	129.3	1	0.33	-		73.0	14.9	34.6	121.5	2	-
Vitamin A (µg RAE/d)	584	193	119	1429	43	< 0.001	0		560	174	122	1376	48	< 0.001	0		509	212	128	2044	67	0
Vitamin D (µg/d)	9.4	5.2	2.1	29.5	-	-	0		8.8	4.4	2.3	22.6	-	-	0		7.3	3.1	1.8	18.6	-	0
Vitamin E (mg/d)	14.3	36.7	1.9	395.8	-	-	0		8.0	2.2	4.0	15.2	-	-	0		7.3	1.5	3.1	12.4	-	0
Thiamin (mg/d)	2.4	5.6	0.6	46.0	14	< 0.001	-		1.0	0.2	0.6	1.7	51	0.02	-		1.0	0.2	0.5	1.6	63	-
Riboflavin (mg/d)	2.5	3.6	0.5	30.9	7	< 0.001	-		1.4	0.3	0.5	2.3	22	0.02	-		1.3	0.3	0.6	2.5	34	-
Niacin (mgNE/d)¶	23.0	9.4	8.6	75.6	2	0.43	0		19.3	5.0	7.0	36.3	4	0.99	0		18.6	4.7	6.1	34.3	4	0
Vitamin B_6_ (mg/d)	3.7	8.7	0.5	74.1	3	< 0.001	2		1.4	0.4	0.5	2.7	15	0.004	0		1.2	0.3	0.4	2.4	28	0
Vitamin B_12_ (µg/d)	13.1	55.3	1.8	616.7	1	0.79	-		6.9	3.2	1.7	18.1	1	0.79	-		6.2	2.8	1.7	21.0	1	-
Folate (µg/d)	411	153	99	860	3	0.11	0		395	141	101	851	4	0.21	0		354	117	88	877	7	0
Vitamin C (mg/d)	192	205	17	1367	15	0.001	-		125	54	22	326	25	0.25	-		106	39	22	265	31	-
Calcium (mg/d)	590	209	199	1331	52	< 0.001	0		545	175	201	1065	62	0.06	0		490	167	185	1212	72	0
Magnesium (mg/d)	305	92	100	608	34	0.046	-		301	88	101	585	38	0.17	-		280	69	3	13	45	-
Phosphorus (mg/d)	1146	256	471	1894	-		0		1133	251	472	1818	-	-	0		1054	213	119	605	-	0
Iron (mg/d)**	9.1	2.5	4.1	19.7	32	0.01	0		8.8	2.3	4.1	15.7	37	0.08	0		8.1	1.8	500.5	1703.0	46	0
Zinc (mg/d)	9.0	2.0	4.5	14.6	29	0.02	0		8.8	2.0	4.5	14.6	30	0.04	0		8.4	2.0	3.1	17.8	41	0
Copper (mg/d)	1.3	0.3	0.6	2.2	1	0.33	0		1.3	0.3	0.6	2.2	1	0.33	0		1.2	0.3	0.5	2.3	3	0

EAR, Estimated Average Requirement; RAE, retinol activity equivalents; NE, niacin equivalents; UL, Tolerable Upper Intake Level

*Usual energy and nutrient intake was calculated separately by users and non-users using the Multiple Source Method. Usual energy and nutrient intake from total intake among users were calculated based on four-day measurements of energy and nutrient intake from total intake considering intake from fortified foods and dietary supplements. Usual energy and nutrient intake from base diet were calculated based on four-day measurements of energy and nutrient intake from base diet

†Users were who reported the use of at least one fortified food or dietary supplement during the 4-day recording period

‡Total intake is the sum of intake from a base diet, fortified foods, and dietary supplements. Total intake among non-users was the same as intake from base diets

§P-value by chi-squared test for distribution of participants with < EAR in total intake among users and non-users. *P* < 0.05 was considered as significant

||P-value by chi-squared test for distribution of participants with < EAR in intake from base diets among users and non-users. *P* < 0.05 was considered as significant

¶Niacine equivalents = niacin (mg) + protein (mg)/6000

**For women aged 18–64 y, EAR for menstrual women were used as a reference value

None of the participants exceeded the UL of the base diet for any of the nutrients examined. After considering the intake of fortified foods and dietary supplements, 2% of the users of fortified foods and/or dietary supplements exceeded the UL for vitamin B_6_ intake.

### Comparison of food intake

Users of fortified foods and/or dietary supplements had a higher intake of fruits, fish and seafood, and milk and dairy products than non-users (Table 5). However, users consumed more confectionaries than non-users.

**Table 5:** Comparison of food intake (g/d) from base diets between fortified foods and/or dietary supplements users and non-users among 392 Japanese adults (aged 20–69 y)

	Users (*n* = 122)		Non-users (*n* = 270)		P*
	Mean	SD	Mean	SD	
Cereals	433	147	458	152	0.13
Potatoes	47	36	42	32	0.17
Sugars	17	13	15	12	0.16
Pulses	60	61	52	46	0.13
Nuts	4	9	3	7	0.25
Fruits	79	82	55	63	0.002
Total vegetables	281	130	259	119	0.11
Fish and seafood	79	53	66	43	0.02
Meat	91	53	94	53	0.56
Eggs	38	22	39	22	0.69
Milk and dairy food products	119	104	88	87	0.002
Fat and oils	21	10	21	10	0.91
Confectioneries	46	34	39	35	0.04
Alcoholic beverages	125	271	139	249	0.62
Tea and coffee	644	384	584	376	0.15
Sweetened beverages	51	100	37	85	0.18
Seasonings	124	81	115	83	0.34

*P value by t-test. *P* < 0.05 was considered as significant

## Discussion

In this study of Japanese adults, users of fortified foods, dietary supplements, or both tended to have a higher intake of dietary fibre, vitamins, minerals, fruits, fish and seafood, and milk and dairy food products than non-users. In addition, users had a lower prevalence of inadequate intake of the six vitamins and minerals than non-users in the base diet excluding fortified foods/dietary supplements. After considering the intake of fortified foods and/or dietary supplements, the prevalence of inadequate intake decreased by > 10% for five vitamins and minerals among users. This is the first study to examine the contribution of fortified foods and dietary supplements to the total nutrient intake and the adequacy of nutrient intake in Japanese adults. No previous study reported the contribution of fortified foods and dietary supplements to the total nutrient intake nor the adequacy of nutrient intake among Asian countries including Japan. Considering the increased consumption of fortified foods and dietary supplements in worldwide, results from this study would be useful for both Japan and other Asian countries.

In the present study, 10% of participants were fortified food users, 24% were dietary supplement users, and 31% were users of either. This prevalence of users was similar to that reported in a previous online survey of Japanese adults aged 20–79 years [3]. However, other studies reported a much higher (55% of males and 61% of females for dietary supplements) [12] and lower (1.4–2.8% for dietary supplements and < 1% for fortified foods) [13] prevalence of users in Japanese than the prevalence in this study. This inconsistency may be due to differences in the characteristics of the participants and definitions of users. A high prevalence was reported in a study of middle-aged or older adults using a questionnaire asking about the use of dietary supplements in the previous year [12]. A low prevalence was reported in a study that used a 1-day dietary record [13]. It might be reasonable that our estimate ranged in the middle of the previous estimates [12, 13], considering the age of the participants and the method (i.e. 4-day dietary record) used in this study. It should be noted that the assessment in this short period could be highly affected by several factors such as sample size, sample selection, and intake frequency of fortified foods and dietary supplements. A previous US study concluded that combining a questionnaire to assess dietary supplement use with at least one 24-h recall was the most comprehensive method for assessing the prevalence of dietary supplement intakes [30]. Therefore, further studies that combine questionnaires and dietary records are required. Moreover, studies are needed to develop and evaluate the validity of questionnaires to assess supplement use in the Japanese population.

Similar to previous studies [5, 10], users of fortified foods and/or dietary supplements were older than non-users in this study. In contrast, there was no difference in the distribution of sex or educational level. A possible reason for the similarity in participants’ characteristics in this study is that most of them worked at welfare facilities. Regarding dietary habits, users in this study tended to have a favourable dietary intake from the base diet. Similar to our results, adult American users of mineral-containing supplements had a high intake of calcium, iron, magnesium, zinc, and phosphorus [14]. Supplement use was associated with dietary patterns of high fruit and vegetable consumption among French adults [15]. In addition, among Korean adults, supplement users tended to have breakfast regularly, restrict snacking, and limit drinking [16]. Thus, fortified foods and dietary supplements may tend to be consumed by individuals with a low requirement for intake of these products. Additionally, there may be reverse causality in associations between use of fortified foods and dietary supplement and users’ characteristics. It is possible that some participants were more inclined to improve their dietary habits and use fortified foods and dietary supplements due to their worsening health.

Among users, the contribution of fortified food use (0.094–3.9% on average) and dietary supplements (0.01–20.6%) was lower than that observed in studies from European countries [4, 8, 11]. For example, among children and adults in the United Kingdom, fortified foods contributed to the total intake of vitamins and minerals in the range of 2% (potassium, selenium, and zinc] to 15% (folate), except for iodine (0%), and dietary supplements contributed to the total intake in the range of 3% (calcium and copper) to 40% (vitamin B_12_), except for potassium (0%) [4]. Despite the small contribution to the total nutrient intake in this study, the intake of fortified foods and dietary supplements contributed to the intake adequacy for some nutrients among users. The prevalence of inadequate thiamine, riboflavin, and vitamin B_6_ intakes decreased significantly among users after considering the intake of fortified foods and dietary supplements. A large decrease in the prevalence of inadequacy among users was also observed for vitamin C and calcium after considering intake from fortified foods and dietary supplements, which might be due to the high contributions from fortified foods in some participants. Thus, the intake of fortified foods and dietary supplements may contribute to the improved intake of some vitamins and minerals, especially vitamins Bs (including thiamine, riboflavin, and vitamin B_6_) and C and calcium. When comparing dietary intake with UL, 2% of users exceeded the UL for vitamin B_6_ in this study. Similar results were reported in a previous study that compared nutrient intake from dietary supplements with UL in the Japanese population [12]. In the US, the prevalence of exceeding the UL was higher than that in this study [14]. These results suggest that supplement use may increase the risk of exceeding the UL. In our study, there is a possibility that only days with low intake of a certain nutrient were selected for the diet record by chance, because the survey period was only four days. Therefore, it is important to note that the risk of excess intake of some nutrients might have underestimated. Altogether, fortified foods and/or dietary supplements should be used appropriately by individuals in need.

This study had several limitations. First, the number of users and intake of fortified foods and dietary supplements were assessed using a 4-day dietary record. Consequently, participants whose consumption frequency was less than 1 time per 4-day recording period might likely have been classified as non-users. In addition, the use of fortified foods was not identified from dietary records if the participants did not record the brand names of the foods. Moreover, some participants may not have recorded their dietary supplement use because the primary aim of this study was to estimate the intake of sodium and potassium. Thus, the prevalence of fortified food or dietary supplement users as well as their nutrient intake may be underestimated. Second, this study was based on a relatively small, non-random sample. Participants may have been health-conscious because they worked at welfare facilities. Therefore, further studies using nationally representative samples are required. Third, although there could have been some seasonal effects on food choice [31] among participants, which could have influenced the use of dietary supplements and fortified foods, dietary information was collected only in winter. Therefore, data collection over several seasons is desirable in future studies. Fourth, the estimate of nutrient intake is prone to misreporting, particularly because of changes in dietary habits during the assessment period [32]. However, as reported in a previous study [33], the proportions of under- and over-reporters of energy intake evaluated in terms of the ratio of reported energy intake to basal metabolic rate based on the Goldberg cutoff method [34] were relatively small (3.6% and 2.3%, respectively). Thus, we consider the potential impact of energy misreporting on the present findings to be minimal. Finally, the relatively small sample size (*n* = 392) limited the power to detect statistically significant moderate differences between users and non-users. In addition, the estimated usual intake distributions of nutrients and foods are uncertain because of small sample sizes [35]. Our sample size had sufficient power (> 0.80) to detect a mean difference in the intake amount between the two groups, assuming that the mean difference was 10 g/day, the sample weight was 3:7, and the expected standard deviation was 30 (calculated by the PROC POWER procedure using SAS). However, the power to detect a difference is insufficient (< 0.8) when the expected standard deviation increases. Although significant differences in nutrient intake were observed between users and non-users, further studies with large sample sizes are required.

## Conclusions

In conclusion, fortified food and/or dietary supplement users had better nutrient intake profiles than non-users in Japan when nutrient intake from fortified foods and/or dietary supplements was not considered. Among users, fortified foods and dietary supplements contributed to the adequate intake of some vitamins and minerals, especially thiamine, riboflavin, vitamin B_6_, vitamin C, and calcium. Nevertheless, there was a risk of exceeding the UL when using fortified food and dietary supplements among some users of vitamin B_6_. These results can be used to design strategies for improving micronutrient intake in Japan and could be the foundation for future research with more nationally representative samples.

## Electronic supplementary material

Below is the link to the electronic supplementary material.

Supplementary Material 1

Supplementary Material 2

## Data Availability

The dietary intake datasets analysed during the current study are not publicly available due to data protection regulations but are available from the corresponding author on reasonable request. The nutrient content dataset regarding fortified foods and dietary supplements in Japanese, which was generated and analyzed for the current study, is available from the corresponding author on reasonable request.
