# Supplementary material for: Contribution of fortified foods and dietary supplements to total nutrient intakes and their adequacy in Japanese adults
Source: BMC Nutr. 2024 Sep 27;10:125. doi: 10.1186/s40795-024-00935-w (PMC11438197; doi:10.1186/s40795-024-00935-w)
Supplement: Supplementary file 1 — Supplementary Material 1 [file 40795_2024_935_MOESM1_ESM.docx]

Supplemental Table 1. Definition of food groups

| Food group | Representative foods |
| --- | --- |
| Cereals | Rice; oats; barley; rye; corn; wheat; bread; Japanese noodles; Chinese noodles; macaroni and spaghetti; White table bread; soft rolls; croissants; wheat flour; fu (gluten products); chio tzu pastry (skin of dumpling); pizza crust; cornflakes |
| Potatoes | Potatoes; sweet potatoes; Japanese yam; French fries |
| Sugars | Brown sugar lump; soft sugars; honey; strawberry jams; orange marmalade |
| Pulses | Adzuki beans; kidney beans; peas; soyabeans and their products (tofu; natto) |
| Nuts | Almonds; chestnuts; walnuts; peanuts |
| Fruits | Fresh and canned fruits; excluding fruits juice |
| Total vegetables | Asparagus; okra; pumpkin; tomatoes; Chinese chive; carrot; parsley; green sweet peppers; broccoli; spinach; Brussels sprouts; Salted pickles of Japanese apricots; salted pickles of cucumber; pickles of Japanese radish; pickles of eggplants; Turnip; cauliflower; cabbage; cucumber; ginger; zucchini; celery; Japanese radish (root); onion; eggplant; Chinese cabbage; winter mushrooms; shiitake; bunashimeji (brown beech mushroom); common mushrooms; purple laver; ma-kombu (kelp); hijiki; wakame |
| Fish and seafoods | Horse mackerel; sardines; salmons; tunas; shrimps; crabs; squids; fish paste products |
| Meat | Beef; pork; ham; bacon; chicken |
| Eggs | Japanese quail’s eggs; hen’s eggs |
| Milk and dairy food products | Ordinary liquid milk; whole milk powder; cream; yoghurt; natural cheese; processed cheese |
| Fat and oils | Lard; butters; olive oil; sesame oil; rapeseed oil; margarines; shortening; mayonnaise |
| Confectioneries | *Manju* (baked or steamed dough stuffed with filling); amedama (drops); rice crackers; bean jam bun; sponge cake; doughnuts; biscuits; milk chocolate; chewing gums; ice cream |
| Alcoholic beverages | Sake; beer; wine; whisky; mirin |
| Tea and coffee | Green teas; oolong tea; black tea; coffee; cocoa |
| Sweetened beverages | Fruits juice; lactic acid bacteria beverages; fruit-flavoured and coloured drink; cola drink |
| Seasonings | Worcester sauces; soya sauces; common salt; vinegars; soup stocks; dressings; miso; roux |

Supplemental Table 2. Reference values of Dietary Reference Intakes for Japanese 2020

|  | Males | | | | Females | | | |
| --- | --- | --- | --- | --- | --- | --- | --- | --- |
|  | 18–29 y | 30–49 y | 50–64 y | 65–74 y | 18–29 y | 30–49 y | 50–64 y | 65–74 y |
| Estimated Average Requirement | |  |  |  |  |  |  |  |
| Protein (g/d) | 50 | 50 | 50 | 50 | 40 | 40 | 40 | 40 |
| Vitamin A (μg RAE/d) | 600 | 650 | 650 | 600 | 450 | 500 | 500 | 500 |
| Thiamin (mg/d) | 1.2 | 1.2 | 1.1 | 1.1 | 0.9 | 0.9 | 0.9 | 0.9 |
| Riboflavin (mg/d) | 1.3 | 1.3 | 1.2 | 1.2 | 1 | 1 | 1 | 1 |
| Niacin (mgNE/d) | 13 | 13 | 12 | 12 | 9 | 10 | 9 | 9 |
| Vitamin B_6_ (mg/d) | 1.1 | 1.1 | 1.1 | 1.1 | 1 | 1 | 1 | 1 |
| Vitamin B_12_ (μg/d) | 2 | 2 | 2 | 2 | 2 | 2 | 2 | 2 |
| Folate (μg/d) | 200 | 200 | 200 | 200 | 200 | 200 | 200 | 200 |
| Vitamin C (mg/d) | 85 | 85 | 85 | 80 | 85 | 85 | 85 | 80 |
| Calcium (mg/d) | 650 | 600 | 600 | 600 | 550 | 550 | 550 | 550 |
| Magnesium (mg/d) | 280 | 310 | 310 | 290 | 230 | 240 | 240 | 230 |
| Iron (mg/d) | 6.5 | 6.5 | 6.5 | 6 | 8.5* | 9* | 9* | 5 |
| Zinc (mg/d) | 9 | 9 | 9 | 9 | 7 | 7 | 7 | 7 |
| Copper (mg/d) | 0.7 | 0.7 | 0.7 | 0.7 | 0.6 | 0.6 | 0.6 | 0.6 |
| Tolerable Upper Intake Level | |  |  |  |  |  |  |  |
| Vitamin A (μgRAE/d) | 2700 | 2700 | 2700 | 2700 | 2700 | 2700 | 2700 | 2700 |
| Vitamin D (μg/d) | 100 | 100 | 100 | 100 | 100 | 100 | 100 | 100 |
| Vitamin E (mg/d) | 850 | 900 | 850 | 850 | 650 | 700 | 700 | 650 |
| Niacin (mgNE/d) | 300 | 350 | 350 | 300 | 250 | 250 | 250 | 250 |
| Vitamin B_6_ (mg/d) | 55 | 60 | 55 | 50 | 45 | 45 | 45 | 40 |
| Folate (μg/d) | 900 | 1000 | 1000 | 900 | 900 | 1000 | 1000 | 900 |
| Calcium (mg/d) | 2500 | 2500 | 2500 | 2500 | 2500 | 2500 | 2500 | 2500 |
| Phosphorus (mg/d) | 3000 | 3000 | 3000 | 3000 | 3000 | 3000 | 3000 | 3000 |
| Iron (mg/d) | 50 | 50 | 50 | 50 | 40 | 40 | 40 | 40 |
| Zinc (mg/d) | 40 | 45 | 45 | 40 | 35 | 35 | 35 | 35 |
| Copper (mg/d) | 7 | 7 | 7 | 7 | 7 | 7 | 7 | 7 |

RAE, retinol activity equivalents; NE, niacin equivalents.

*Values for menstrual women.
